# Experimental validation of proton boron capture therapy for glioma cells

**DOI:** 10.1038/s41598-023-28428-z

**Published:** 2023-01-24

**Authors:** Tatiana Shtam, Vladimir Burdakov, Alina Garina, Luiza Garaeva, Nhan Hau Tran, Andrey Volnitskiy, Eva Kuus, Dmitry Amerkanov, Fedor Pack, Georgy Andreev, Andrey Lubinskiy, Konstantin Shabalin, Nicolay Verlov, Evgeniy Ivanov, Victor Ezhov, Dmitry Lebedev, Andrey L. Konevega

**Affiliations:** 1 Petersburg Nuclear Physics Institute Named By B.P. Konstantinov of National Research Centre “Kurchatov Institute”, Leningradskaya Oblast, Mkr. Orlova Roshcha 1, Gatchina, Russian Federation 188300; 2 National Research Center “Kurchatov Institute”, Akademika Kurchatova Pl. 1, Moscow, Russian Federation 123182; 3 Institute of Cytology of Russian Academy of Sciences, St. Petersburg, Russian Federation; 4 Peter the Great St.Petersburg Polytechnic University, Politehnicheskaya 29, St. Petersburg, Russian Federation; 5 Proton Therapy Center MIBS, St. Petersburg, Russian Federation

**Keywords:** Radiotherapy, Cancer models, Radiotherapy

## Abstract

Proton boron capture therapy (PBCT) has emerged from particle acceleration research for enhancing the biological effectiveness of proton therapy. The mechanism responsible for the dose increase was supposed to be related to proton-boron fusion reactions (^11^B + *p* → 3*α* + 8.7 MeV). There has been some experimental evidence that the biological efficiency of protons is significantly higher for boron-11-containing prostate or breast cancer cells. The aim of this study was to evaluate the sensitizing potential of sodium borocaptate (BSH) under proton irradiation at the Bragg peak of cultured glioma cells. To address this problem, cells of two glioma lines were preincubated with 80 or 160 ppm boron-11, irradiated both at the middle of 200 MeV beam Spread-Out Bragg Peak (SOBP) and at the distal end of the 89.7 MeV beam SOBP and assessed for the viability, as well as their ability to form colonies. Our results clearly show that BSH provides for only a slight, if any, enhancement of the effect of proton radiation on the glioma cells in vitro. In addition, we repeated the experiments using the Du145 prostate cancer cell line, for which an increase in the biological efficiency of proton irradiation in the presence of sodium borocaptate was demonstrated previously. The data presented add new argument against the efficiency of proton boron capture therapy when based solely on direct dose-enhancement effect by the proton capture nuclear reaction, underlining the need to investigate the indirect effects of the secondary alpha irradiation depending on the state and treatment conditions of the irradiated tissue.

## Introduction

Proton therapy (PT) has been applied for many years for the treatment of patients with different types of cancer. PT is characterized by a well-defined dose deposition in tissue with a sharp increase of the absorbed dose at a specific depth which depends on the initial beam energy (Bragg Peak)^1^. Beyond the Bragg Peak region, where the major part of the beam energy is absorbed, the dose drops sharply within next few millimeters of tissue. Such a dose distribution makes it possible to achieve a higher absorbed dose in the tumor region keeping the radiation damage to the surrounding tissue at acceptable level. Proton therapy could be particularly appropriate for the treatment of brain tumors, as it allows for the precise delivery of the radiation to the specific region of brain^2^.

Gliomas are the most common primary brain tumors with an incidence rate of about 6 cases per 100,000 population per year^3^. The median survival of patients with low-grade glioma can range from 6 to more than 15 years^3^, whereas the median survival for patients with glioblastoma, the most frequent and malignant high-grade gliomas, is often reported as few months^3–5^. The dosimetric advantages and the safety of PT for treatment of gliomas have been previously reported^6,7^. Several tumor-specific drugs are under development to improve the overall effectiveness of currently employed radio therapy, including PT, towards high-grade gliomas^8,9^, underlining the overall interest in targeted therapies aimed at improving the therapeutic ratio by increasing the selectivity of radio therapy toward the tumor cells and thus decreasing its adverse effects on the normal tissues^9^.

One of the emerging treatment modalities designed to improve the therapeutic ratio is boron neutron capture therapy (BNCT) which is based on neutron capture by non-radioactive ^10^B nuclei, a nuclear reaction process that can bring about a very significant dose enhancement in tissue enriched with ^10^B isotope. According to the early clinical studies, BNCT has the potential to become effective as a targeted therapy^10^. Being initially limited by the need of usage of research nuclear reactors as the neutron source, nowadays BNCT approach is boosted by the availability of specialized compact neutron source specially designed for medical purposes^11^ Several investigations are under way for the use of BNCT in high grade glioma treatment^12,13^. One of the critical components for successful deployment of BNCT is the choice of compounds for ^10^B delivery to the tumor that would meet the requirements for high tumor uptake, low normal tissue uptake, low toxicity and rapid clearance after treatment^14–16^. Currently the two major compounds used for this purpose are sodium borocaptate ((B_12_H_11_SH)Na_2_), BSH) and boronophenylalanine (C_9_H_12_BNO_4_, BPA)^10^. Approval of these boron-containing drugs in clinical trials of BNCT could give a clear advantage for their use in other types of radiotherapy.

Proton boron capture therapy (PBCT) was proposed as a potential cancer treatment method a few decades ago. In PBCT the dose enhancement effect was assigned to the reaction of nuclear fusion between ^11^B nuclei and the incident beam protons (^11^B + *p* → ^8^Be + *α → *3*α* + 8.7 MeV). Emission of the high Linear Energy Transfer (LET) α-particles by proton-boron fusion reaction, that occurs primarily in the Bragg peak region and causes irreparable clustered DNA damages, was expected to increase the effectiveness of destruction of the tumor cell. There are two experimental reports showing a significant increase in the effectiveness of proton beam irradiation of ^11^B-containing cancer cells^17,18^. However, other works containing simulation and analytical calculations seem to indicate that the total number of the generated alpha particles cannot explain the experimental results^19,20^. Thus, the reported evidence for the possibility to use of boron-containing compounds for the dose enhancement in PT as well as the possible mechanisms of their radio-sensitizing effect remain contradictory and, in the view of a very limited number of studies on PBCT, call for a further investigation.

In the present study we have examined the possibility to enhance biological effects of proton irradiation in boron-containing cultured glioma cells.

## Results

We examined the radiosensitizing effect of sodium borocaptate for proton beam irradiation applied to two glioma cell lines (A172 and Gl-Tr), as well prostate cancer cells Du145, for which such an effect of BSH was demonstrated earlier^17,18^. The boron-11 concentration in most experiments was 80 ppm, and the periods of cell incubation with BSH were 7 h and 18 h. After incubation with BSH the cells were irradiated with 89.7 MeV clinical proton beam, positioning the cells at the distal end of Spread-Out Bragg Peak (SOBP) (Supl. Figure 1). An additional experiment was carried out with irradiation of cells with a 200 MeV proton beam, positioning the cells in the middle of the SOBP (Supl. Figure 1). These incubation points and irradiation conditions were selected based on the earlier reports demonstrating increased cell death under proton irradiation in the presence of different boron-containing compounds^17,18,21^.

**Figure 1:**
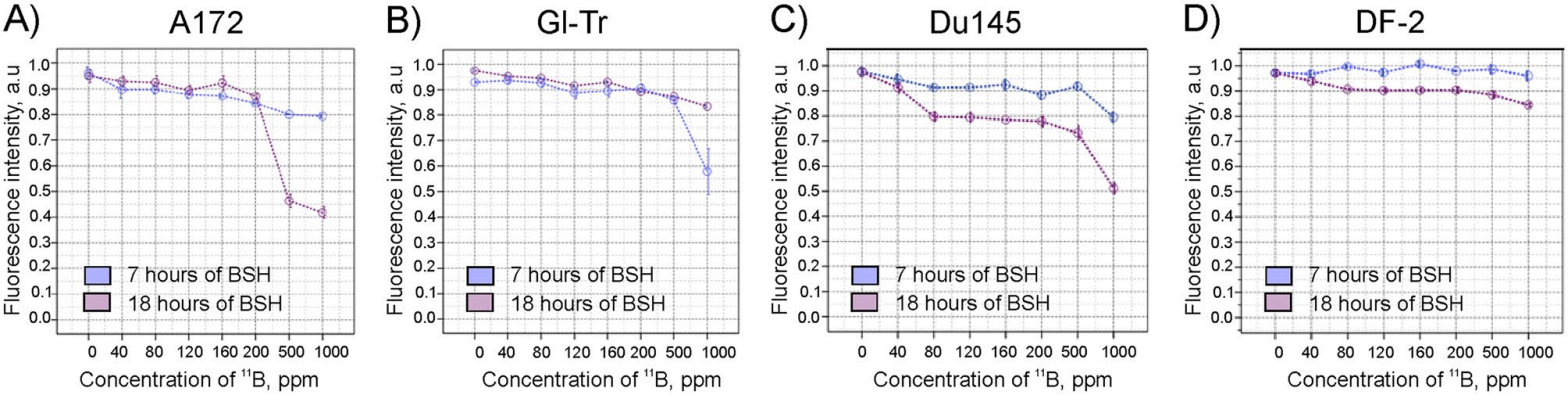
Cytotoxicity of sodium borocaptate (BSH) for A172 (**A**), Gl-Tr (**B**) glioma cells, Du145 prostate cancer cells (**C**), and DF-2 fibroblasts of human skin (**D**). The cytotoxic effects of BSH at 0—1000 ppm of ^11^B concentration have been studied by the AlamarBlue cell viability assay after 7 h or 18 h of incubation.

### Cytotoxicity of sodium borocaptate for glioma cells

To determine the threshold toxic concentration of ^11^B, glioma cells, Du145 cells, as well as fibroblasts of human skin, DF-2, were incubated in the presence of BSH for 7 h and 18 h. The cytotoxic effects of BSH at 40–1000 ppm of ^11^B concentration have been studied by the AlamarBlue cell viability assay. The cytostatic effect of sodium borocaptate on glioma cells, as well as Du145 prostate cancer cells, is manifested at concentrations from 500 ppm of ^11^B and higher (Fig. 1A–C). For normal cells, DF-2 fibroblasts, the cytotoxic effect was not observed over the entire range of analyzed boron concentrations (Fig. 1D). No significant decrease in the survival rate of all studied cell types was observed at 80–160 ppm boron concentration range used in the study.

### Cell cycle parameters of glioma cells in the presence of sodium borocaptate

**Figure 2:**
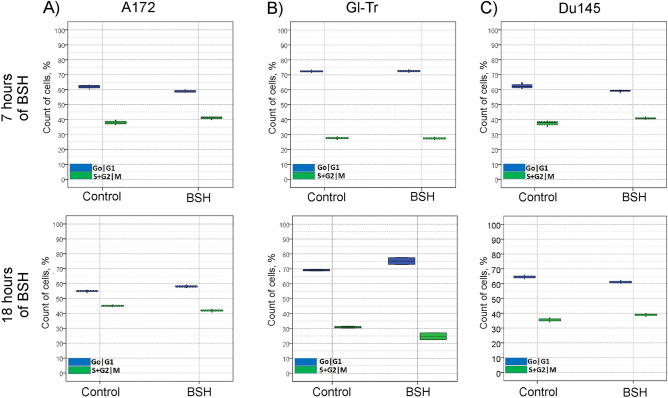
Distribution of A172 (**A**), Gl-Tr (**B**) glioma cells, and Du145 prostate cancer cells (**C**) over the phases of the cell cycle after incubation with sodium borocaptate (BSH) at a boron-11 concentration of 80 ppm for 7 h (up panels) or 18 h (bottom panels).

Measurements of cell cycle parameters of A172 cells during incubation for 7 h (*p* = 0.12) or 18 h (*p* = 0.507) with BSH at a concentration of 80 ppm showed a typical pattern of cell distribution along the cell cycle, recorded in control cells (Fig. 2A). For Gl-Tr cells, a very slight increase in the number of cells in the G0/G1 phase of the cell cycle (*p* = 0.05) was observed during 18 h of incubation with sodium borocaptate (Fig. 2B). Thus, in the analyzed glioma cells, there is no significant effect of sodium borocaptate on the distribution of the phases of the cell cycle. For prostate cancer Du145 cells, no significant changes were observed in the parameters of the cell cycle during incubation of cells in the presence of BSH for both 7 h (*p* = 0.507) and 18 h (*p* = 0.261) (Fig. 2C). The Mann – Whitney test was used to assess the differences between groups (with/without BSH). Level of significance was set at *p* < 0.05. Examples of cell distribution by cell cycle phases in the presence of sodium borocaptate are shown in Supplementary Fig. S2.

### Effect of preincubation with 80 ppm boron-11 as part of BSH on the sensitivity of glioma cells to proton irradiation at the Bragg peak

**Figure 3:**
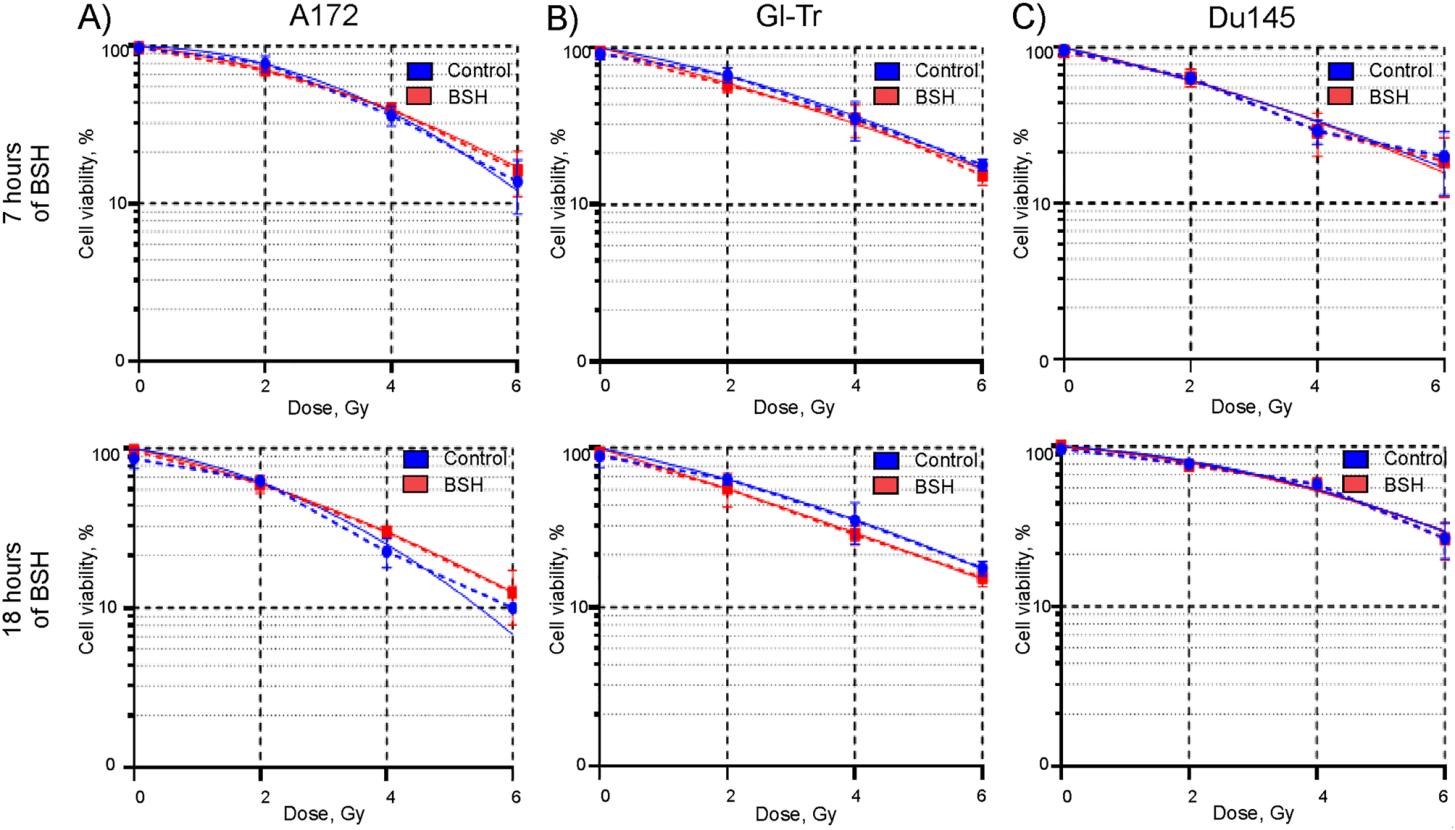
Effect of BSH on cell viability after exposure to Bragg peak protons at different absorbed dose. Summary plots for AlamarBlue cell viability assays: (**A**) A172 cells, (**B**) Gl-Tr cells, (**C**) DU145 cells (dashed lines). The cells were incubated in a medium containing 80 ppm of boron-11 for 7 h (up panels) or 18 h (bottom panels) and then irradiated by 89.7 MeV clinical proton beam in a dose range of 0–6 Gy. Data were fitted with a linear-quadratic function of the radiation dose (solid lines). Each plot summarizes the results of three independent irradiation experiments.

Next, we investigated whether the presence of sodium borocaptate increased the sensitivity of glioma cells to proton irradiation. Cells were incubated in a medium containing 80 ppm of boron-11 for 7 h or 18 h and then irradiated at the distal end of SOBP of 89.7 MeV clinical proton beam in a dose range of 0–6 Gy. Irradiation was carried out in the presence of boron-11 in the cell culture medium. The efficiency of irradiation was determined using both the AlamarBlue Assay and the Clonogenic assay. Cell viability was fitted with a linear-quadratic function of the radiation dose. In addition, irradiation dose for half-maximal inhibition of cell viability (IC_50_) and a dose-modifying factor for 20% cell survival (DMF_20_) in the presence of borocaptate, as compared to irradiation alone, were determined. The Mann – Whitney test was used to assess the differences between groups (with/without BSH) and one sample t-test—to evaluate difference DMF_20_ values from 1. Evaluation of the effectiveness of radiosensitization with sodium borocaptate of glioma cells, carried out using the AlamarBlue Cell Viability Reagent, did not demonstrate any significant effect from the presence of boron in the cell culture medium before and during irradiation (Fig. 3A,B). Fitting parameters, irradiation dose for half-maximal inhibition of viability, and dose-modifying factor for 20% survival of Gl-Tr cells with irradiation alone or after incubation with sodium borocaptate for 7 h are presented in Table 1. A similar lack of sensitizing effect was observed for Du145 cells (Fig. 3C). The dose-modifying factors (DMF_20_) for Du145 cells irradiated after incubation with BSH for 7 and 18 h were 1.03 ± 0.01 (*p* = 0.535) and 0.95 ± 0.06 (*p* = 0.492) respectively. The values of the determined survival parameters for all studied cell lines summarized in Supplementary Tables 1–3.

**Table 1:** Parameters for the linear-quadratic fit of cell viability curve Y(X) = exp (–αX–βX^2^), irradiation dose for half-maximal inhibition of viability (IC_50_), and dose-modifying factor for 20% survival (DMF_20_) of Gl-Tr cells with proton irradiation alone (Control) or after incubation with 80 ppm of boron-11 (BSH) for 7 h.

	α	β	IC_50_, Gy	DMF_20_
Control	0.16 ± 0.05	0.022 ± 0.002	2.4 ± 0.4	1.02 ± 0.03 (*p* = 0.566)
BSH	0.24 ± 0.05	0.009 ± 0.001	2.1 ± 0.3 (p = 0.268)	

**Figure 4:**
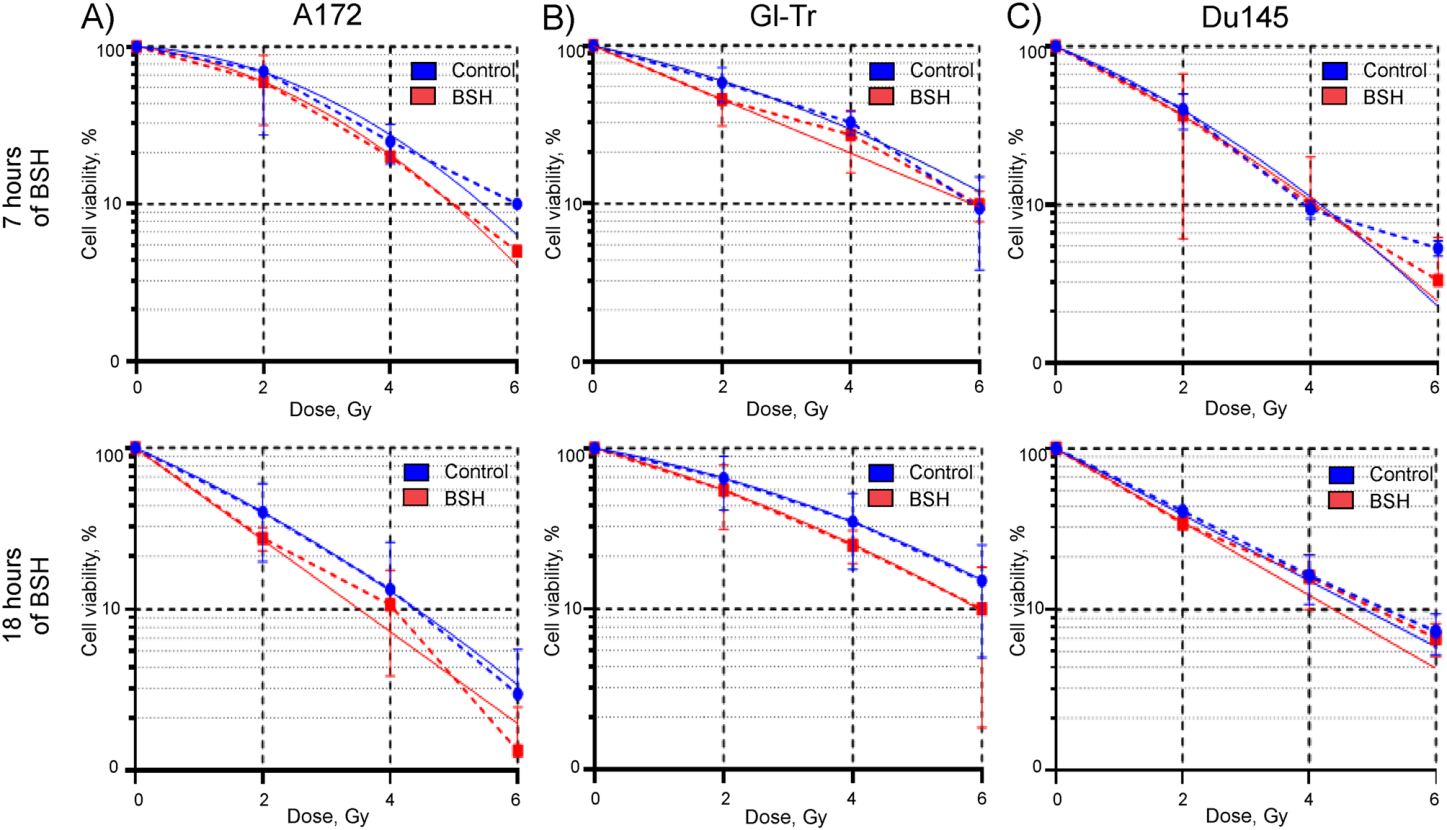
Effect of BSH on cell colony formation after exposure to Bragg peak of 89.7 MeV clinical proton beam. Summary plots for colony assays: (**A**) A172 cells, (**B**) Gl-Tr cells, (**C**) Du145 cells (dashed lines). Data were fitted with a linear-quadratic function of the radiation dose (solid lines) with the parameter β constrained to non-negative values. The cells were incubated in a medium containing 80 ppm of boron for 7 h (up panels) or 18 h (bottom panels) and then irradiated by 89.7 MeV clinical proton beam in a dose range of 0–6 Gy. After treatment, 10^3^ cells were seeded out to form sufficiently large clones consisting of 50 or more cells. Colony formation assay was performed in 10–14 days. Each plot summarizes the results of three independent experiments.

The AlamarBlue assay is commonly used to quantify the number of live cells in a sample, and to monitor cell viability / cytotoxicity. But only a fraction of live seeded cells retains the capacity to produce colonies after irradiation. Clonogenic assay is the method of choice to determine cell reproductive death after treatment with ionizing radiation. Therefore, at the next step of the study, we tested the ability of glioma cells incubated with 80 ppm of boron-11 to form colonies after irradiation with an 89.7 MeV clinical proton beam at the distal end of Bragg peak. For cells of both glioma lines we observed a very slight and not significant effect of reducing the number of viable colonies in the samples incubated and irradiated in the presence of sodium borocaptate (Fig. 4A,B). For example, for Gl-Tr cells, the irradiation doses corresponding to 50% survival of colonizing cells were 1.9 ± 0.8 and 1.4 ± 0.6 (*p* = 0.275) for irradiation alone and for irradiation of cells preincubated with BSH for 7 h. At the same time, the dose-modifying factor, DMF_20_ for these cells was 1.32 ± 0.15 (*p* = 0.07). This weak effect was not reproduced stably in independent experiments and did not depend on the time of incubation with the BSH (Fig. 4A,B). Moreover, for Du145 cells, we did not observe a significant effect of sodium borocaptate introduced into the culture medium on the formation of viable colonies after clinical proton beam irradiation (Fig. 4C). DMF_20_ for Du145 cells preincubated with BSH for 7 and 18 h were 0.99 ± 0.12 (*p* = 0.741) and 1.25 ± 0.02 (*p* = 0.05), respectively. The values of the determined survival parameters for all studied cell lines summarized in Supplementary Tables 1–3. Examples of colony assays for proton boron capture treatment are shown in Supplementary Fig. S3.

**Figure 5:**
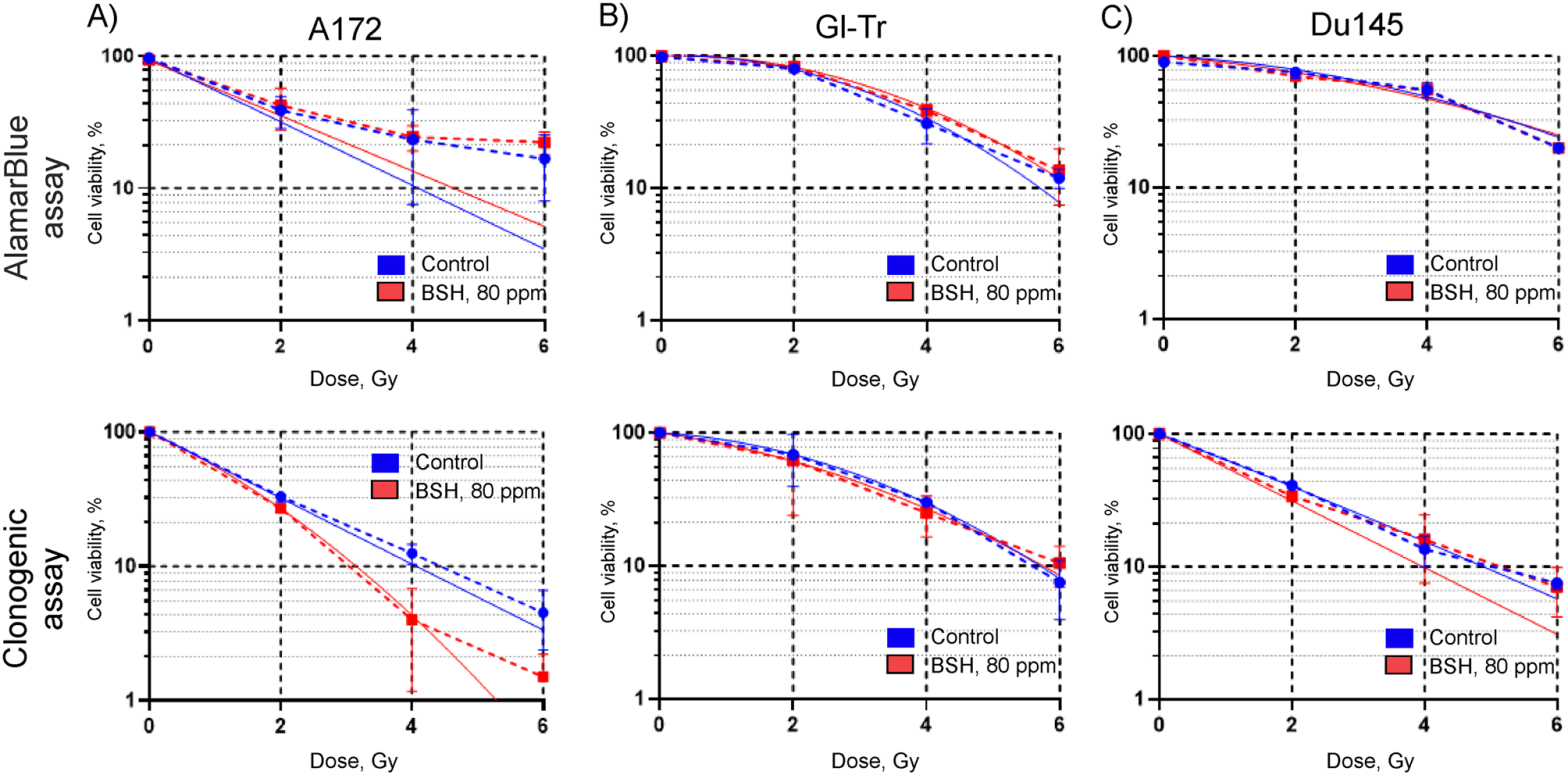
Comparison of cell survival under combined exposure to 80 ppm boron-11 and irradiation at the middle position of Spread-Out Bragg Peak of 200 MeV proton beam. Summary plots for AlamarBlue (up panels) and colony forming assays (bottom panels): (**A**) A172 cells, (**B**) Gl-Tr cells, (**C**) Du145 cells. The cells were incubated in a medium containing 80 ppm of boron-11 for 18 h and then irradiated by 200 MeV proton beam in a dose range of 0–6 Gy. Data were fitted with a linear-quadratic function of the radiation dose (solid lines) with the parameter β constrained to non-negative values.

Experiments to determine the survival of the cells and their ability to form colonies after incubation with BSH were also carried out using a 200 MeV proton beam. The parameters of cell irradiation, as well as the conditions of cell incubation with boron-11, were chosen similar to our previous study, in which we observed the sensitizing effect of sodium tetraborate (Na_2_B_4_O_7_) during proton irradiation of glioma cells^21^. Cells were incubated with 80 ppm of boron-11 for 18 h, transferred to test tubes in a complete culture medium without BSH, irradiated at the middle position of SOBP (Suppl. Fig. S1), and then analyzed for viability by AlamarBlue and colony forming assay. A comparative analysis of the metabolic activity of cells after irradiation with a 200 MeV proton beam did not reveal any radiosensitizing effect of boron-11 for both glioma cells and prostate cancer cells (Fig. 5, upper panels). DMF_20_ for A172, Gl-Tr and DU145 cells preincubated with sodium borocaptate were less than unity 0.98 ± 0.29 (*p* = 0.948), 0.92 ± 0.08 (*p* = 0.02) and 0.98 ± 0.31 (*p* = 0.468), respectively. When analyzing the ability of cells to form colonies after proton irradiation, a slight decrease in the number of colonies of A172 cells preincubated with BSH was observed compared with only irradiated cells (Fig. 5A, bottom panel). The determined IC_50_ for these cells without and with sodium borocaptate was 0.75 ± 0.30 and 0.50 ± 0.30 (*p* = 0.05), and DMF_20_ was 1.20 ± 0.01 (*p* = 0.05) (Suppl. Table S1). No significant radiosensitizing effect of boron-11 was observed for the second glioma cell line (Fig. 5B, bottom panel). Prostate cancer cells formed the same number of colonies after irradiation with a 200 MeV proton beam, both after cell incubation with and without sodium borocaptate (Fig. 5C, bottom panel). DMF_20_ for Gl-Tr and DU145 cells preincubated with sodium borocaptate were 1.02 ± 0.12 (*p* = 0.415) and 1.19 ± 0.04 (*p* = 0.144), respectively (Suppl. Tables S2,S3). Examples of colony assays for proton boron capture treatment at the middle position of SOBP of 200 MeV proton beam are shown in Supplementary Fig. S4.

### Possibilities for increasing the biological effect of the sodium borocaptate on the survival of cells when irradiated with clinical protons at the Bragg peak

**Figure 6:**
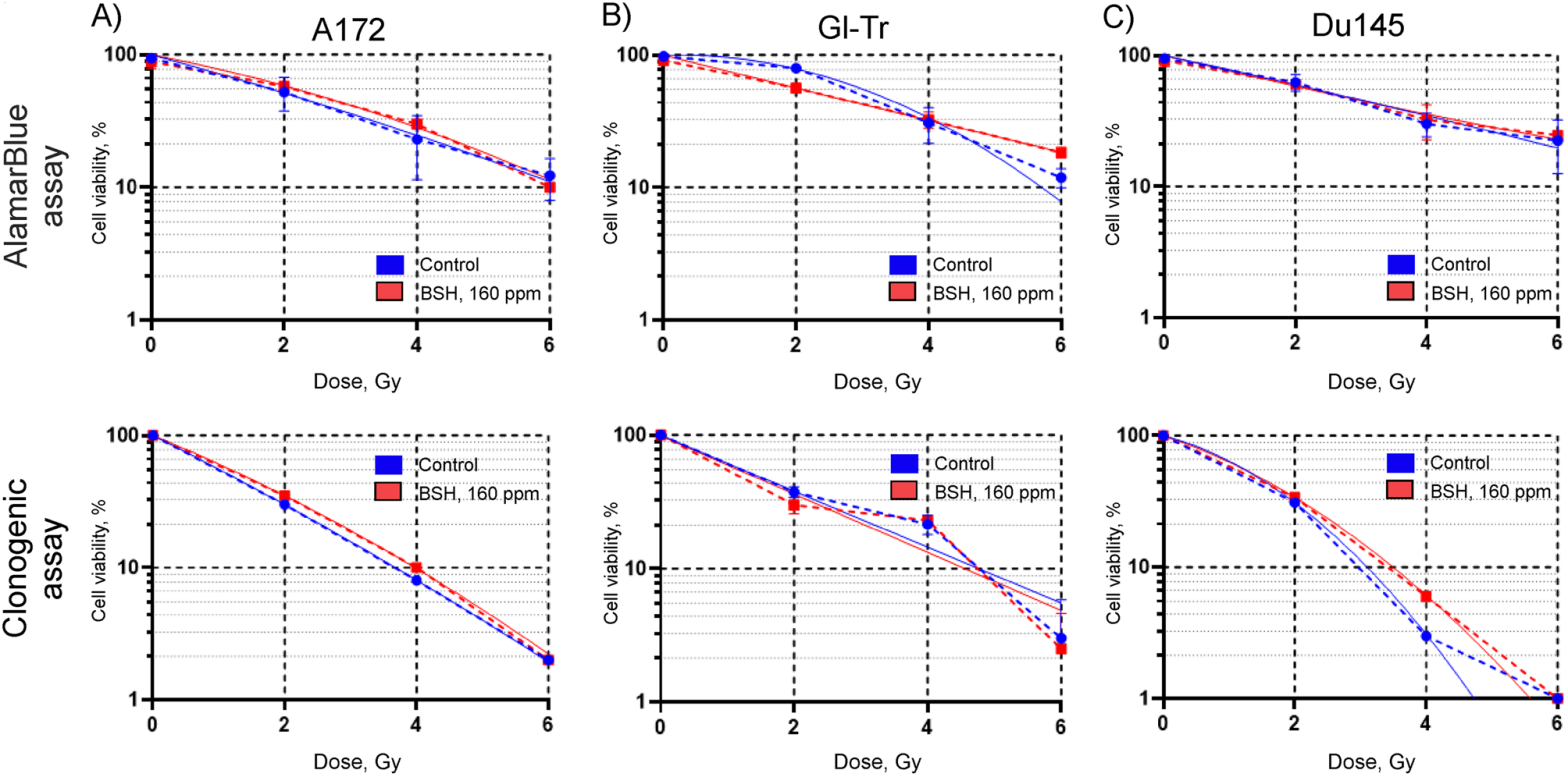
Cell viability after incubation of cells with 160 ppm of boron-11 and exposure to Bragg peak of 89.7 MeV clinical proton beam. Summary plots for AlamarBlue (up panels) and colony forming assays (bottom panel): (**A**) A172 cells, (**B**) Gl-Tr cells, (**C**) Du145 cells. The cells were incubated in a medium containing 160 ppm of boron-11 for 18 h and then irradiated at the distal position of 89.7 MeV clinical SOBP in a dose range of 0–6 Gy. Experimental data (dashed lines) were fitted with a linear-quadratic function of the radiation dose (solid lines) with the parameter β constrained to non-negative values.

Despite the high content of boron atoms per single BSH molecule ((B_12_H_11_SH)Na_2_), sodium borocaptate has a disadvantage for clinical usage for BNCT of gliomas, namely, poor cellular uptake by brain tumors^15,22^. To test the hypothesis about the possible insufficiency of boron in the culture medium of glioma cells to obtain the radiosensitizing effect of BSH for proton boron capture therapy, we increased the concentration of boron-11 up to 160 ppm. We did not observe any sensitizing effect when the amount of BSH was doubled during incubation and 89.7 MeV clinical proton beam irradiation of glioma cells (Fig. 6A,B). Moreover, we also did not observe any effect of boron proton capture therapy for Du145 prostate cancer cells (Fig. 6C), for which the expected effect was described earlier^17,18^. The values of the determined survival parameters for all studied cell lines are summarized in Supplementary Tables 1–3. Examples of colony forming assays for 7 h of incubation of cells with 160 ppm boron-11 and subsequent clinical proton beam irradiation are shown in Supplementary Fig. S3.

**Figure 7:**
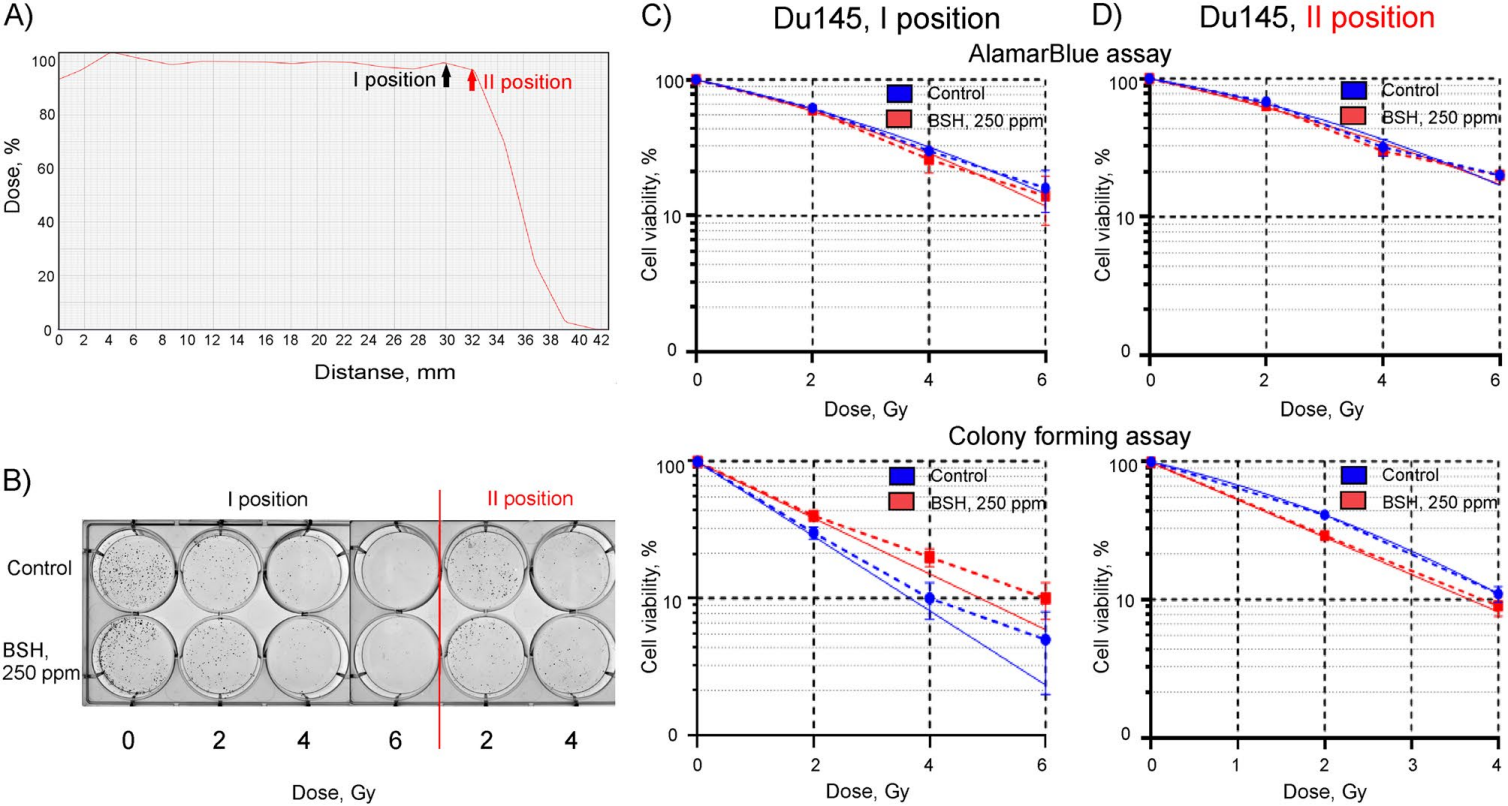
Effect of BSH on cell survivability after exposure to Bragg peak protons at different absorbed dose. (**A**) Dose profile for irradiation of cells with an 89.7 MeV clinical proton beam. In the majority of experiments, the cells were positioned at a depth of 30 mm (I position), and in some—at a depth of 32 mm (II position) along clinical proton SOBP. (**B**) Examples of colony forming assays for 7 h of incubation of Du145 cells with BSH (0 ppm (Control) or 250 ppm of boron-11) and proton beam irradiation at I position or II position of proton SOBP. (**C,D**) Plots for AlamarBlue (up panels) and colony forming assays (bottom panels) of Du145 cells. The cells were incubated in a medium containing 250 ppm of boron-11 and then irradiated by proton beam in a dose range of 0–6 Gy at (**C**) I position or (**D**) II position of SOBP of 89.7 MeV clinical proton beam.

Since the cross section of the ^11^B + *p* → 3 *α* + 8.7 MeV nuclear reaction increases with a decrease in the average proton beam energy, we tried to increase the biological efficiency of boron proton capture by positioning the cells in the most distal part of the Bragg peak. To test the hypothesis, the Du145 cells were positioned at a depth of 30 mm (I position tested in all previous experiments), and at a depth of 32 mm (II position) along the 89.7 MeV clinical proton SOBP, at the distal end of the Bragg peak (Fig. 7A). In addition, in these experiments we used a triple concentration of borocaptate corresponding to 250 ppm of boron-11. Example of colony forming assays for 7 h of incubation of Du145 cells with BSH and subsequent proton beam irradiation along the proton SOBP is shown in Fig. 7B. No dose enhancement effect was observed when cells were irradiated in the presence of BSH both at the position I of the proton beam and at position II, at the distal end of the SOBP (Fig. 7C,D). The calculated DMF_20_ for cell irradiation at position I with or without sodium borocaptate was close to 1 when using the AlamarBlue metabolic test and less than 1 for the clonogenic assay. The similar factors for the irradiation position II were indistinguishable from 1 (Suppl. Table S3). Summarizing the results, we found that irradiation-induced cellular lethality was either not enhanced by pre-treatment of cells with BSH, or the observed enhancement was rather small and not statistically significant.

## Discussion

A method to increase the biological effectiveness of proton beam is of high interest in radiation oncology. In thefirst publication on proton boron capture in radiation therapy authors used Monte Carlo simulations and showed an enhanced high dose in the areas where the boron uptake coincided with the Bragg peak^23^. In this work the increase of the maximum dose level was reported at about 50% for 80 MeV proton energy beam. Also, the increase of the maximum dose level of PBCT was reported at 192.4% for 75 MeV beam^24^. The mechanism responsible for a higher dose was suggested to be related to proton-boron fusion reactions, leading to the production ofhigh Linear Energy Transfer (LET) α-particles.

There are a few other studies which used Monte Carlo simulations, but which produced highly divergent results on the potentially achievable boron proton capture dose based on using different proton energies and ^11^B concentrations^20,25,26^. These later simulations confirm that the number of induced reactions on boron-11 in proton therapy is negligible. For example, according to the results of Tayebeh A et al. the total dose of alpha particles generated from boron proton capture for 70–85 MeV proton beam is piddling, even in the situation that the entire tumor region was considered full of boron-11^20^. Also, according to simulations of Mazzone et al. at 80 ppm concentration of ^11^B and 45–60 MeV proton energies, the effect of the p + ^11^B → 3α reaction is negligible and the dose related to this reaction several orders of magnitude lower than the dose delivered by the primary proton beam^25^. In fact, both results indicate that the reaction probability is too low to bear any effect in the therapy.

On the basis of these considerations, it seems highly unlikely that the reported decrease in cellular survival in the presence of BSH^17,18^ is related with the boron proton capture. Another explanation for the described radiosensitizing effect of boron-11 may be the biochemical properties of the boron-containing compound. For example, according to our previous results the radiosensitizing effect of boron-11 on the viability of the cancer cells subjected to proton irradiation was detected in vitro using two human malignant glioma cell lines, and sodium tetraborate (Na_2_B_4_O_7_) as a boron containing substance^21^. But the fact that we observed a similar radiosensitizing effect of sodium tetraborate on A172 cells when they were subjected to gamma-radiation indicated that the decrease in the survival rate of cells irradiated after pre-incubation with the boron containing compound is likely due to its pharmacological and chemical properties rather than an increase in the absorbed dose due to ^11^B + p → 3α nuclear reaction^21^. In this work we have also tested the possibility of increasing the biological effects of gamma radiation by the presence of sodium borocaptate and observed no effect (Suppl. Fig. S5, Suppl. Tables S1–S3).

In this study, we examined the radiosensitivity of two glioma cell lines by proton beam irradiation in the presence of sodium borocaptate. Our results clearly show that BSH, being non-toxic to the normal morphology DF-2 cells and to the glioma cells at the concentrations used, produces little, if any, enhancement of the effect of proton radiation on the glioma cell lines in vitro. Since the additional dose enhancement generated by boron proton capture depends on the proton energy and intracellular ^11^B concentration, we have tested various variants of incubation of glioma cells with BSH, and also carried out a series of irradiation on two cyclotrons with different energies of the proton beam. Fitting parameters and dose-modifying factor for 20% survival for colony forming assays of glioma cells with irradiation alone or after incubation with sodium borocaptate are presented in Table 2. Despite the fact that the dose-modifying factor in some experiments was greater than one, in all experiments the radiosensitizing effect of sodium borocaptate was not statistically significant and did not depend on the time of preincubation with BSH (7 or 18 h), concentration of boron-11 (80 or 160 ppm), or on the initial energy of the proton beam (89.7 or 200 MeV).

**Table 2:** Parameters for the linear-quadratic fit of clonogenic survival data Y(X) = exp (–αX–βX^2^), and dose-modifying factor for 20% survival (DMF_20_) of Du145, A172, and Gl-Tr cells with proton irradiation alone (Control) or after incubation with boron-11 (BSH).

Radiation	Time of incubation	BSH concentration	Sample	DU145			A172			Gl-Tr		
				α	β	DMF_20_	α	β	DMF_20_	α	β	DMF_20_
89.7 MeV	18 h	80 ppm	Control	0.45 ± 0.01	0	1.25 ± 0.02 (p = 0.05)	0.41 ± 0.25	0.027 ± 0.008	1.28 ± 0.07 (p = 0.113)	0.17 ± 0.02	0.025 ± 0.004	1.18 ± 0.12 (p = 0.126)
			BSH	0.57 ± 0.01	0		0.65 ± 0.03	0		0.26 ± 0.02	0.022 ± 0.005	
		160 ppm	Control	0.21 ± 0.03	0.10 ± 0.03	0.99 ± 0.08 (p = 0.868)	0.57 ± 0.13	0.025 ± 0.005	0.8 ± 0.5 (p = 0.374)	0.48 ± 0.03	0	1.12 ± 0.11 (p = 0.178)
			BSH	0.10 ± 0.03	0.13 ± 0.05		0.47 ± 0.05	0.013 ± 0.002		0.51 ± 0.03	0	
	7 h	80 ppm	Control	0.32 ± 0.04	0.07 ± 0.02	0.99 ± 0.12 (p = 0.741)	0.05 ± 0.03	0.07 ± 0.02	1.1 ± 0.3 (p = 0.227)	0.21 ± 0.14	0.025 ± 0.004	1.32 ± 0.15 (p = 0.07)
			BSH	0.45 ± 0.06	0.028 ± 0.001		0.12 ± 0.02	0.07 ± 0.03		0.39 ± 0.17	0	
		250 ppm position I	Control	0.63 ± 0.02	0	0.75 ± 0.08 (p = 0.05)						
			BSH	0.48 ± 0.02	0							
		250 ppm position II	Control	0.34 ± 0.06	0.059 ± 0.027	1.17 ± 0.12 (p = 0.868))						
			BSH	0.62 ± 0.03	0							
200 MeV	18 h	80 ppm	Control	0.45 ± 0.02	0	1.19 ± 0.04 (p = 0.144))	0.57 ± 0.03	0	1.20 ± 0.01 (p = 0.05)	0.07 ± 0.02	0.058 ± 0.006	1.02 ± 0.12 (p = 0.415)
			BSH	0.58 ± 0.02	0		0.53 ± 0.10	0.065 ± 0.006		0.16 ± 0.08	0.041 ± 0.009	
Gamma	18 h	\\\]80 ppm	Control	0.036 ± 0.007	0.026 ± 0.003	1.00 ± 0.02 (p = 0.353)	0.18 ± 0.02	0.019 ± 0.002	1.00 ± 0.13 (p = 0.369)	0.08 ± 0.01	0.023 ± 0.003	1.05 ± 0.13 (p = 0.305)
			BSH	0.014 ± 0.005	0.029 ± 0.004		0.13 ± 0.06	0.028 ± 0.001		0.07 ± 0.01	0.027 ± 0.003	

Given the published data^12,15,22^, it is possible that the apparent lack of radiosensitizing effect of BSH upon glioma cell irradiation with protons could be explained by the ineffective uptake of this compound by the studied glioblastoma cells. Testing this hypothesis, we repeated all experiments using the Du145 prostate cancer cell line, for which an increase in the biological efficiency of proton irradiation in the presence of sodium borocaptate was demonstrated^17,18^. In these two experimental studies the authors obtained a first pre-clinical demonstration of PBCT at the relatively low-energy clinical proton beamline (62 MeV) and at the high-energy proton beamline (250 MeV) reporting a significant reduction in the colony-forming ability of prostate cancer Du145 cells irradiated in the presence of the boron carrier BSH^17,18^. In both experiments, the authors preincubated cells for 6–8 h with 80 ppm of boron-11 and irradiated cells at various depths along the two clinical SOBPs, observing the radiosensitizing effect of BSH at the mid- and distal SOBP positions^17,18^.

In this study, we used the previously reported experimental conditions^17,18^: we pre-incubated Du145 prostate cancer cells with BSH (boron-11 concentration 80 ppm) for 7 h; irradiated with a 200 MeV proton beam, positioning the cells at the middle SOBP position; irradiated with 89.7 MeV clinical proton beam, positioning the cells at the distal end of SOBP. The effectiveness of the combined exposure was analyzed using both the AlamarBlue cell viability assay and the Clonogenic assay. Fitting parameters and dose-modifying factor for 20% survival for colony forming assays of Du145 cells with irradiation alone or after incubation with sodium borocaptate under various experimental conditions are summarized in Table 2. In all those experiments, proton irradiation in the presence of BSH did not appear to reduce Du145 cell viability or clonogenic survival to the degree comparable to that reported in the works^17,18^. Moreover, neither an increase in the concentration of boron-11 in the culture medium (160 or 250 ppm), nor an increase in the incubation time with sodium borocaptate (18 h) or the positioning of the cells at the most distal end of the clinical SOBP did not increase the sensitizing effect of BSH.

Based on the published experimental data and the results of calculations and simulations^17,18,20,21,25,26^, our results appear to indicate that the mechanisms of the radiosensitizing effects of boron during proton irradiation involves a complex process that is not limited to the dose enhancement by the alpha-particles produced in ^11^B + *p* → 3*α* nuclear reaction. As already indicated, the additional dose produced by this reaction is not sufficient to bring to any substantial cell damage at any reasonable BSH concentrations^25^. Moreover, considering a relative sparseness of the nuclear reaction events that follows from the simulation and the short range of the alpha particles released, these particles appear to directly affect only a small fraction of the irradiated cells. Our results therefore seem to support the hypothesis that radiosensitizing effect of BSH could be to the large extent attributed to the indirect effects of the alpha irradiation^18^, such as radiation induced bystander effects^27–29^. These effects could largely depend on the state and treatment conditions of the cell culture or irradiated tissue, calling for further studies to establish the applicability of the proton boron capture therapy technique.

## Materials and methods

### Reagents

The following reagents were used in the study: sodium mercaptododecaborate (KATCHEM Ltd.,Czech Republic), AlamarBlue Cell Viability Reagent (Invitrogen, USA), Dulbecco′s Modified Eagle′s Medium/Nutrient Mixture F-12 Ham (DMEM/F12 (1:1) medium (BioloT, Russia)), fetal bovine serum (HyClone, USA). All other reagents were obtained from Sigma-Aldrich (USA).

### Cell lines and cultivation conditions

The work was carried out using two human glioma cell lines: A172- glioma cell line was obtained from the collection of the Institute of Cytology of the Russian Academy of Sciences (St. Petersburg, Russia); Gl-Tr—glioma cell line generated in the Laboratory of Cell Biology (NRC «Kurchatov Institute»-PNPI, Gatchina, Russia)^30^; Prostate cancer cell line Du145 was kindly donated by Dr. A. Malek, N.N. Petrov National Medical Research Center of Oncology, St. Petersburg, Russia. DF2- fibroblasts of the skin from eyelids of an adult donor were obtained from the collection of the Institute of Cytology of RAS and were used as a control in cytotoxicity experiments. The cells were cultured in an atmosphere of 5% CO_2_ at 37 °C in DMEM/F12 (1:1) medium containing L-glutamine, 0.1 mg/ml each of penicillin and streptomycin, and supplemented with 10% fetal bovine serum.

### Analysis of BSH cytotoxicity

To establish cytotoxicity of borocaptate, DF-2, A172, Gl-Tr, and Du145 cells were plated in 96-well tissue culture plate in concentration of 5 × 10^3^ cells per well, incubated for 24 h in complete medium, then the medium was replaced with a medium containing BSH in the concentration range, corresponding to ^11^B of 0 – 1000 ppm. After 18 or 7 h of incubation, the proliferative activity of the cells was tested by the AlamarBlue® Assay. AlamarBlue Cell Viability Reagent was added to the plate according to the manufacturer’s protocol and incubated for two hours. The fluorescence was detected using EnSpire Multimode Plate Reader (PerkinElmer, USA). All experiments were carried out in quintuplicate.

### Cell cycle analysis

For the cell cycle analysis, A172, Gl-Tr, and Du145 cell lines were plated at 4 × 10^5^ cells/well in a six well tissue culture plate, cultured for 24 h, then the medium was replaced with a medium containing BSH (80 ppm of ^11^B). After 18 or 7 h of incubation in the presence of BSH, the cells were trypsinized, washed with phosphate-buffered saline (PBS), and incubated for 10 min in a solution containing Triton X-100 (0.1% solution v/v) and Hoechst 33,342 (10 µg/ml). The fraction of cells in G0/G1, S and G2/M, based on Hoechst 33,342 labelling of DNA content, was determined by flow cytometry (Beckman Coulter, USA). For each sample, 10^4^ events were collected. Experiments were performed in quintuplicate.

### Irradiation

Cell irradiation was performed in two ways. For the first way, two days before irradiation A172, Gl-Tr, and Du145 cells were seeded in T25 flasks at 5 × 10^5^ cells/flask and were cultured for 24 h. Then the cell growth medium was aspirated and replaced with 5 ml of fresh medium (control) or with BSH-containing medium. The working concentration of 80 ppm of ^11^B corresponded to 0.17 mg/ml of BSH. After 18 h of incubation in the presence of ^11^B, the cells were trypsinized, washed with medium, resuspended in complete culture medium without BSH, and counted by LUNA-II™ Automated Cell Counter (Logos Biosystems, South Korea). Then each cell suspension was split into five portions (0.1 ml each), transferred into sterile Eppendorf tubes at 10^5^ cells/tube, and irradiated. Proton irradiations were performed on 1 GeV synchrocyclotron SC-1000 (NRC «Kurchatov Institute»-PNPI, Gatchina, Russia) using Roscosmos test facility (IS OP CDKT.412110.013) equipped with a variable length remote-controlled copper plate degrader and a bending magnet to reduce the energy spread^31^. The degrader length was set to 491 mm to generate a 200 MeV proton beam with an energy spread of approximately 10 MeV. The beam had a round cross-section with diameter of 30 mm (intensity variations not exceeding 10%). The beam was directed into a plexiglas tissue-equivalent phantom, resulting in a Spread-Out Bragg Peak (SOBP) located ca. 22 cm inside the phantom and spanning approximately 25 mm of the phantom depth. The phantom depth was varied by addition of the appropriate number of 2.8 mm plexiglass plates. The position of SOBP was determined by a series of dose rate measurements using Unidos Webline clinical dosimeter, which was also used for the dose calibration. The dose rate variations within SOBP did not exceed 10%. The proton beam was calibrated before each experiment. The dose profile is shown in Supplementary Fig. S1A. Cell samples were positioned in the phantom near the middle of SOBP and irradiated with increasing doses of 0.5–6 Gy. After irradiation the cells were seeded at 1 × 10^4^ cells/well in a 24 well tissue culture plate and incubated at normal conditions during 48 h. The irradiation experiments were carried out in triplicate.

For the second way, two days before irradiation A172, Gl-Tr, and Du145 cells were plated at 4 × 10^4^ cells/well in a six well tissue culture plate, and were cultured for 24 h. Then the cell growth medium was aspirated and replaced with 3 ml of fresh medium (control) or with BSH-containing medium. In most experiments, we used a working concentration of BSH of 80 ppm, but in a number of experiments, concentrations of BSH of 160 ppm and 250 ppm were also used. The working concentrations of 80 ppm, 160 ppm and 250 ppm of ^11^B corresponded to 0.17 mg/ml, 0.34 mg/ml, and 0.53 mg/ml of BSH, respectively. After 18 or 7 h of incubation in the presence of ^11^B, the cells were irradiated in 6-well plates in a medium containing boron-11 with a clinical proton beam at Bragg peak in a dose range of 2–6 Gray. During irradiation, the plates with cells were positioned horizontally in front of the proton beam. Proton irradiation was performed on a Varian ProBeam version 3.5 at the MIBS proton therapy center (Berezin Sergei Medical Institute, St. Petersburg, Russia). Irradiation was carried out with a pencil beam in layers, with the beam proton energy from 89.735 MeV in the first layer to 110.735 MeV in the eighth layer. Spot weight ranged from 0 to 119.98 MU. Irradiation plans were created using Eclipce software, version 13.6. The dose profile is shown in Supplementary Fig. S1B. Water-equivalent plates 30 mm thick were placed above the samples to shift the Bragg peak to the cell layer. In most experiments, the cells were positioned at a depth of 30 mm, and in several—at a depth of 32 mm (Fig. 7A). One hour after irradiation, the culture medium in the plates was replaced with complete culture medium and the cells were incubated under standard conditions for 48 h. All irradiations with a clinical proton beam were carried out in triplicate.

To test whether the physical nuclear reaction ^11^B(p,3α) affects the cancer cell death by proton beam irradiation, cell lines were also exposure to 80 ppm boron-11 and gamma radiation at different absorbed dose. After 18 h of incubation in the presence of ^11^B, the cells were trypsinized, washed with medium, resuspended in complete culture medium without BSH, and counted by LUNA-II™ Automated Cell Counter (Logos Biosystems, South Korea). Then each cell suspension was split into five portions (0.1 ml each), transferred into sterile Eppendorf tubes at 10^5^ cells/tube, irradiated with graded doses 0–8 Gy using ^60^Co γ-ray source "Issledovatel" (NRC «Kurchatov Institute»-PNPI, Gatchina, Russia).

### Cell viability and colony formation assays after irradiation

For cell viability assay irradiated and control cells were passaged at a concentration of 1 × 10^3^ cells/well in 96 well tissue culture plate in five replicates of each experimental point. To determine the survival rate, the AlamarBlue® Assay was applied 6 days after irradiation. AlamarBlue Cell Viability Reagent was added to the plate according to the manufacturer’s protocol and incubated for two hours. The fluorescence was detected using EnSpire Multimode Plate Reader (PerkinElmer, USA).

Clonogenic assay is the method of choice to determine cell reproductive death after treatment with ionizing radiation. After irradiation, the cells were seeded at 1 × 10^3^ cells/well in a six well tissue culture plate*,* to form colonies in 1–2 weeks. Cells were incubated in standard conditions until cells in control dishes have formed sufficiently large clones. Then colonies were fixed with 96% ethanol, stained with crystal violet (0.5% w/v), washed with water, dried in air, photographed and counted by free ImageJ software.

### Statistical analysis

Raphical visualization and statistical analysis were performed using SPSS 22.0 and GraphPad Prism 9.4.0. Dose–response curves were constructed fitting the cell viability values to the linear-quadratic equation Y(X) = exp (–αX–βX^2^) by GraphPad Prism 9.4.0 (nonlinear regression with the parameter β constrained to non-negative values), and α/β parameters were used to calculate Dose-Modifying Factor at 20% survival level (DMF_20_). Experimental data are expressed as the mean ± the standard deviation (SD). To assess differences between groups (with or without BSH), the Mann–Whitney test was used. One sample t-test was used to evaluate difference of DMF_20_ values from 1. Level of significance was set at *p* < 0.05.

## Supplementary Information

Supplementary Information.

## Data Availability

All data generated or analyzed during this study are included in this published article and its supplementary information files.
