# Supplementary material for: Experimental validation of proton boron capture therapy for glioma cells
Source: Sci Rep. 2023 Jan 24;13:1341. doi: 10.1038/s41598-023-28428-z (PMC9873635; doi:10.1038/s41598-023-28428-z)
Supplement: Supplementary file 1 — Supplementary Information. [file 41598_2023_28428_MOESM1_ESM.pdf]

Supplementary Information

Experimental validation of proton boron capture therapy for glioma cells

Tatiana Shtam[1,2,*], Vladimir Burdakov[1,2], Alina Garina[1,2,3], Luiza Garaeva[1,2,3], Nhan Hau Tran[3], Andrey Volnitskiy[1,2], Eva Kuus[1,3,4], Dmitry Amerkanov[1,2], Fedor Pack[1,2], Georgy Andreev[4], Andrey Lubinskiy[4], Konstantin Shabalin[1,2], Nicolay Verlov[1,2], Evgeniy Ivanov[1], Victor Ezhov[1], Dmitry Lebedev[1,2], Andrey L Konevega[1,2,3,*]

[1]Petersburg Nuclear Physics Institute named by B.P. Konstantinov of National Research Centre «Kurchatov Institute», Orlova roscha 1, Gatchina, 188300, Russian Federation
[2] National Research Center "Kurchatov Institute", Akademika Kurchatova pl. 1, 123182, Moscow, Russian Federation
[3]Peter the Great St.Petersburg Polytechnic University, Politehnicheskaya 29, St. Petersburg, Russian Federation
[4]Proton therapy center MIBS, St. Petersburg, Russia

*Correspondence should be addressed to:

Dr. Tatiana Shtam, Shtam_TA@pnpi.nrcki.ru, phone (+7) 921 974 44 34, 188300, Russia, Gatchina, mkr. Orlova roscha 1, Petersburg Nuclear Physics Institute named by B.P.Konstantinov of National Research Center "Kurchatov Institute"

Dr. Andrey L. Konevega, konevega_al@pnpi.nrcki.ru, phone: +7 (81371) 46093,
Petersburg Nuclear Physics Institute named by B.P. Konstantinov of NRC «Kurchatov Institute», 188300, Russia, Leningradskaya Oblast, Gatchina, 1, mkr. Orlova Roshcha.

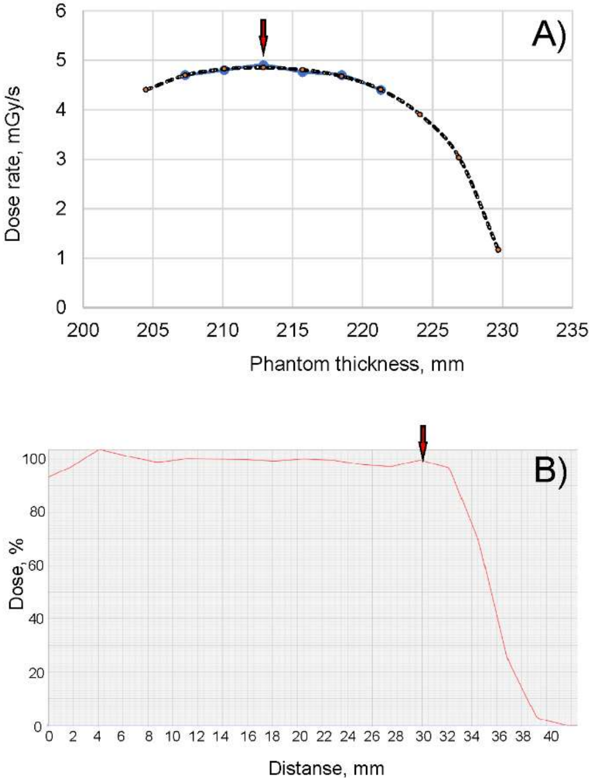

Supplementary figure S1. Dose profile for irradiation of cells with proton beam. (**A**) Dose profile generated by synchrocyclotron SC-1000 (NRC «Kurchatov Institute»-PNPI, Gatchina, Russia). Cells were positioned in the phantom near the middle of Spread-Out Bragg Peak (arrow).
(**B**) Dose profile for irradiation of cells with 89.7 MeV clinical proton beam. Proton irradiation was performed on a Varian ProBeam version 3.5. at the MIBS proton therapy center (Berezin Sergei Medical Institute, St. Petersburg, Russia). The cells were positioned at the distal end of SOBP (arrow).

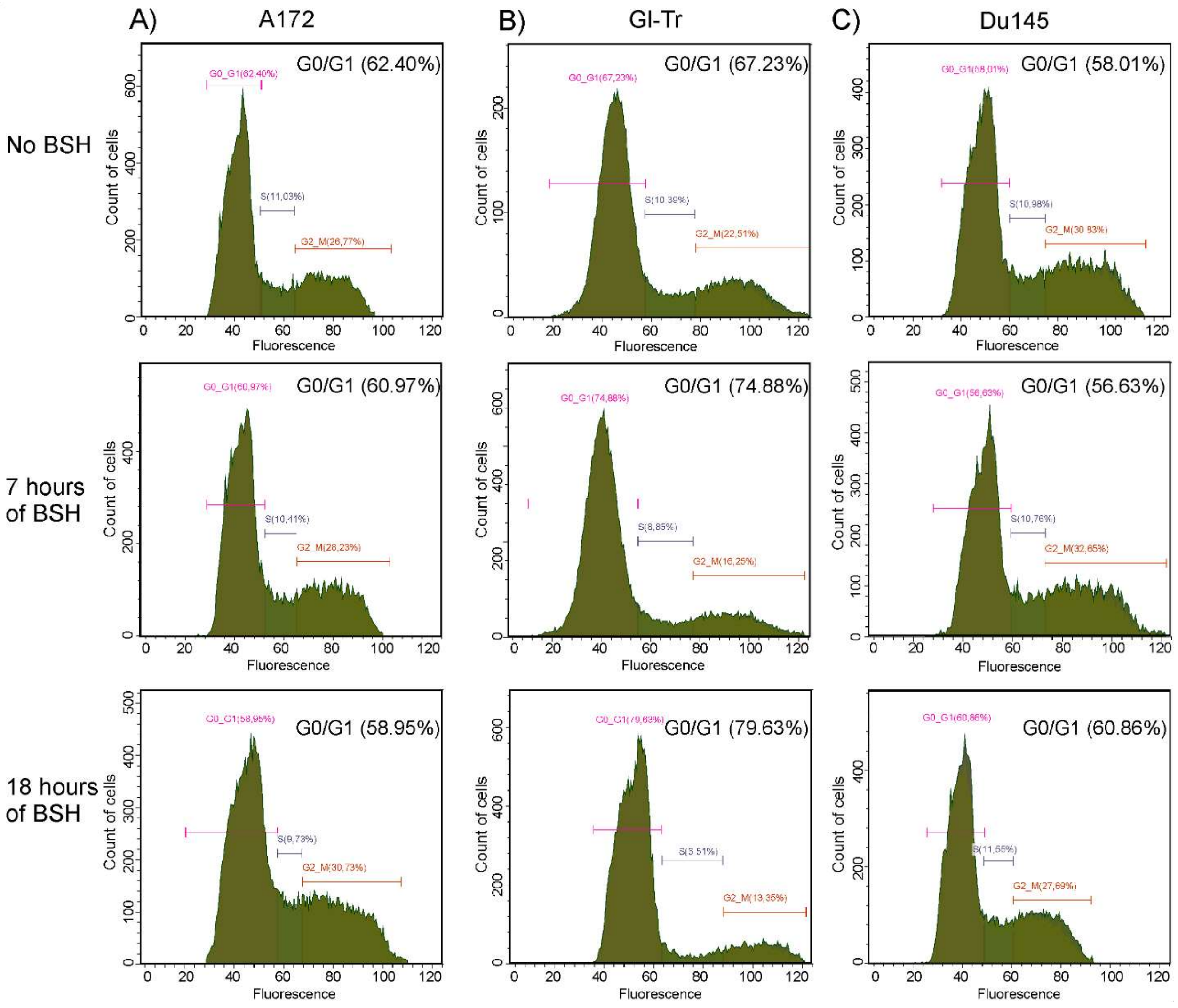

Supplementary figure S2. Effect of sodium borocaptate (BSH) on cycle distribution. A typical cell cycle analysis through Hoechst 33342 staining and following flow cytometry for A172 (**A**), Gl-Tr (**B**) glioma cells and Du145 prostate cancer cells (**C**) after incubation with 80 ppm of boron-11 during 7 hours (middle panels), 18 hours (lower panels) or without it (upper panels).

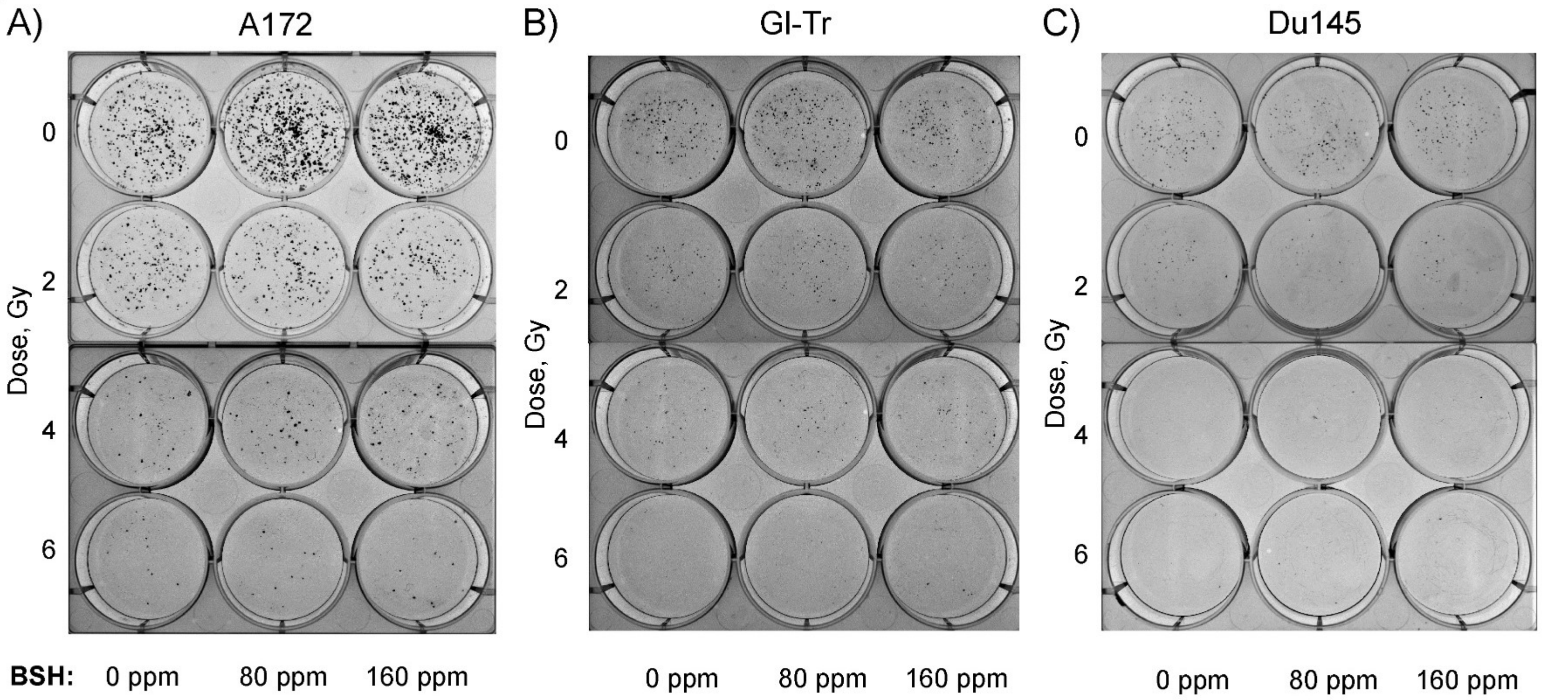

Supplementary figure S3. Effect of sodium borocaptate (BSH) on cell survivability after exposure to Spread-Out Bragg Peak (SOBP) of 89.7 MeV clinical proton beam. Examples of colony forming assays for 7 hours of incubation of cells with BSH: (**A**) A172 cells, (**B**) Gl-Tr cells, (**C**) Du145 cells. The cells were incubated in a medium containing 0 ppm, 80 ppm or 160 ppm of boron-11 for 7 hours and then irradiated in a dose range of 0-6 gray. The cells were irradiated in 6-well plates in a medium containing boron-11 at the distal position of 89.7 MeV clinical SOBP.

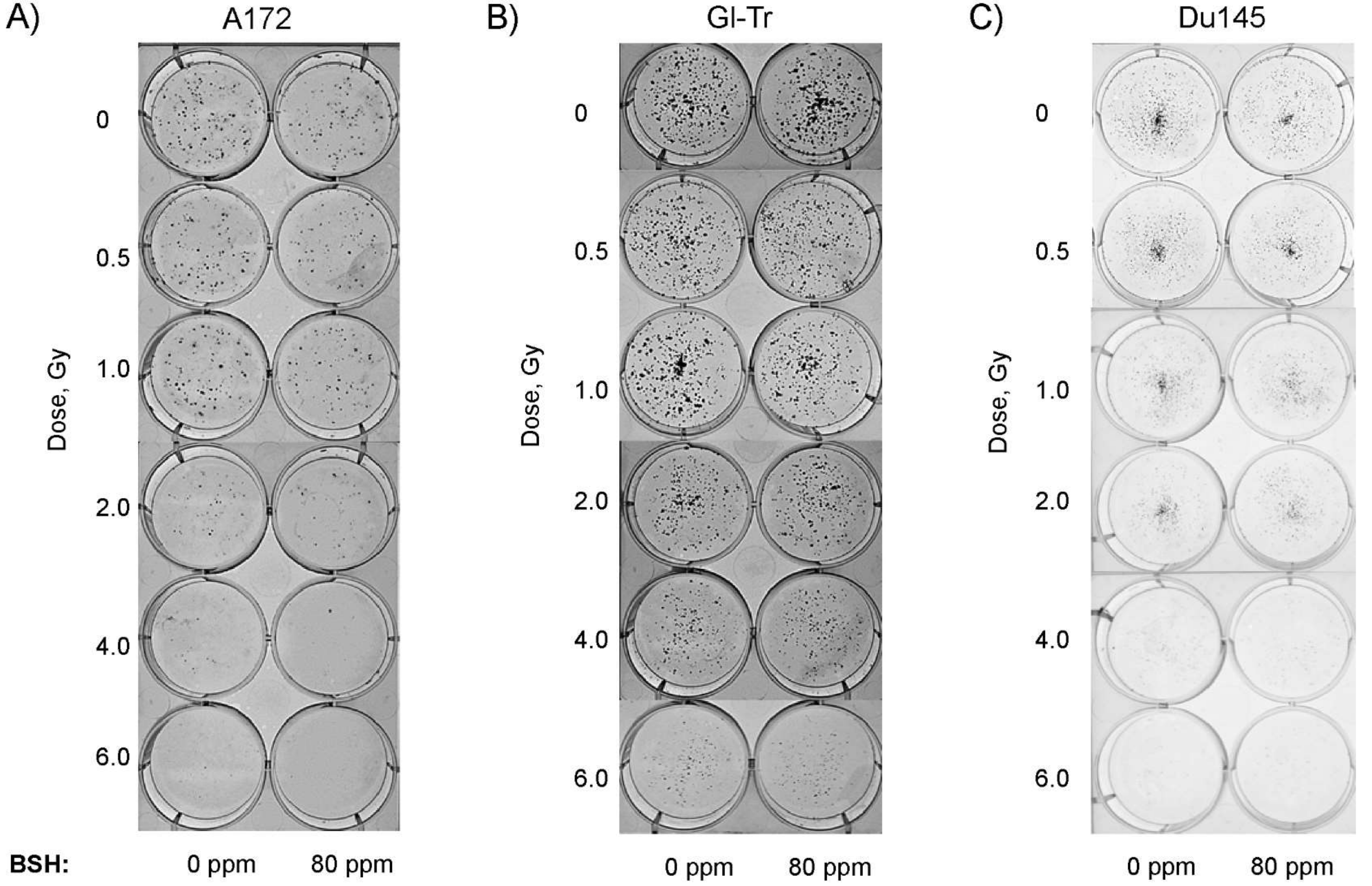

Supplementary figure S4. The ability of cells to form colonies under combined exposure to 80 ppm boron-11 and irradiation at the middle position of Spread-Out Bragg Peak of 200 MeV proton beam. Examples of colony forming assays for 18 hours of incubation of cells with sodium borocaptate (BSH): (**A**) A172 cells, (**B**) Gl-Tr cells, (**C**) Du145 cells. The cells were incubated in a medium containing 0 ppm or 80 ppm of boron-11 for 18 hours and then irradiated with 200 MeV proton beam in a dose range of 0-6 gray.

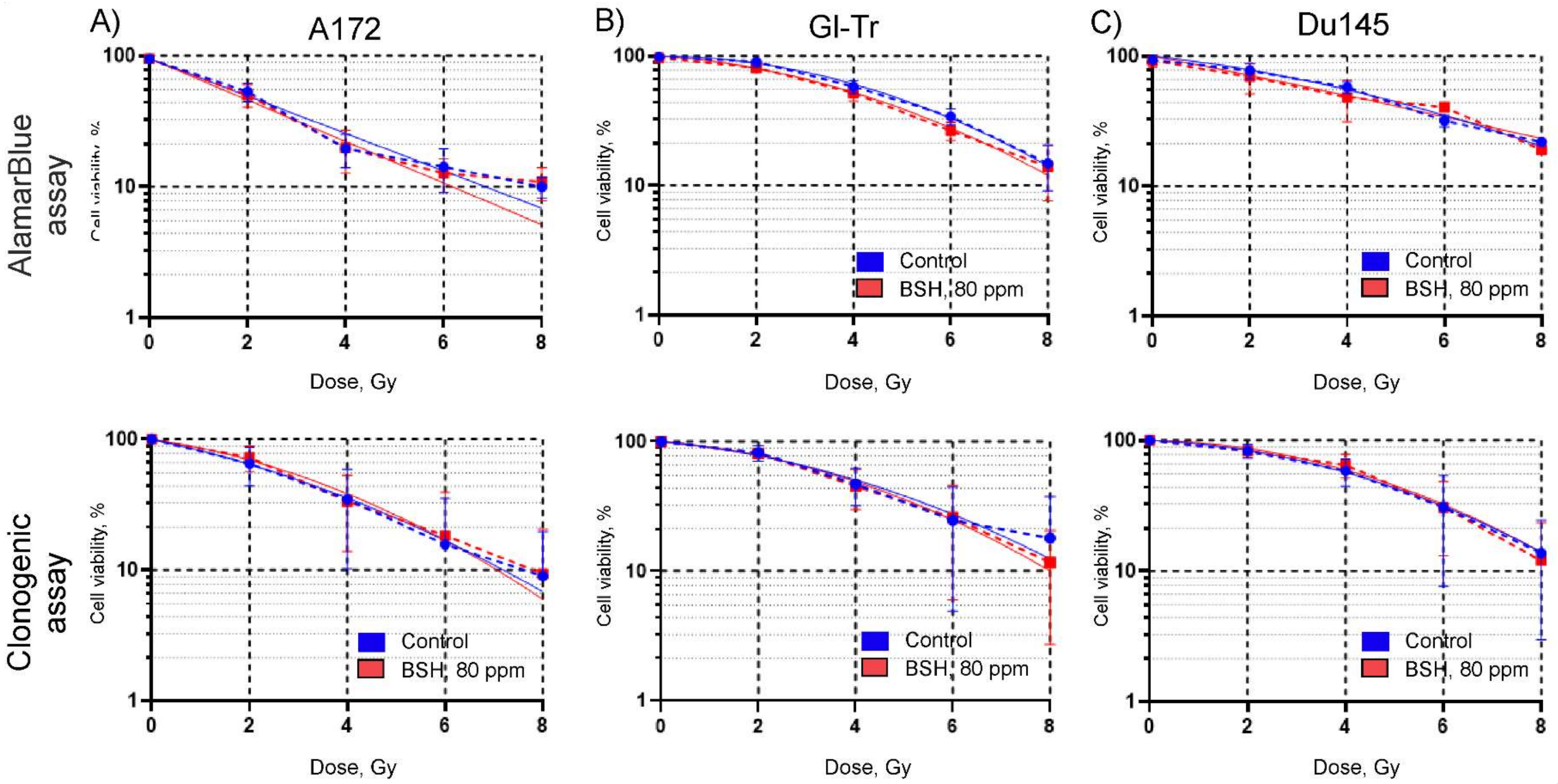

Supplementary figure S5. Comparison of cell survival under combined exposure to 80 ppm boron-11 and gamma radiation at different absorbed dose. Summary plots for AlamarBlue (up panels) and colony forming assays (bottom panels): (**A**) A172 cells, (**B**) Gl-Tr cells, (**C**) Du145 cells. The cells were incubated in a medium containing 80 ppm of boron-11 for 18 hours and then irradiated with graded doses 2-8 Gy using lift-up type ^{60}Co γ-ray source "Issledovatel" (NRC «Kurchatov Institute»-PNPI, Gatchina, Russia). Data were fitted with a linear-quadratic function of the radiation dose (solid lines) with the parameter β constrained to non-negative values.

Cell line	Radiation	Time of incubation	Concentration of BSH	Sample	α		β		IC_{50}, Gy		DMF_{20}	
					AlamarBlue assay	Clonogenic assay	AlamarBlue assay	Clonogenic assay	AlamarBlue assay	Clonogenic assay	AlamarBlue assay	Clonogenic assay
A172	proton beam (89.7 MeV)	18h	80 ppm	Control	0.21±0.07	0.41±0.25	0.025±0.003	0.027±0.008	1.9±0.4	0.9±0.4	0.94±0.09 (p=0.499)	1.28±0.07 (p=0.113)
				BSH	0.23±0.04	0.65±0.03	0.015±0.001	0	2.0±0.2 (p=0.658)	0.8±0.2 (p=0.513)		
			160 ppm	Control	0.32±0.08	0.57±0.13	0.009±0.002	0.025±0.005	1.5±0.2	0.7±0.3	0.94±0.09 (p=0.372)	0.8±0.5 (p=0.374)
				BSH	0.22±0.07	0.47±0.05	0.02±0.01	0.013±0.002	1.7±0.4 (p=0.513)	0.6±0.3 (p=0.275)		
		7h	80 ppm	Control	0.10±0.05	0.05±0.03	0.039±0.003	0.07±0.02	2.4±0.4	2.1±0.9	0.95±0.05 (p=0.364)	1.1±0.3 (p=0.227)
				BSH	0.19±0.05	0.12±0.02	0.018±0.002	0.07±0.03	2.3±0.3 (p=0.658)	1.6±0.7 (p=0.827)		
	proton beam (200 MeV)	18h	80 ppm	Control	0.56±0.19	0.566±0.031	0	0	1.2±0.3	0.75±0.3	0.98±0.29 (p=0.948)	1.20±0.01 (p=0.05)
				BSH	0.48±0.03	0.53±0.10	0	0.065±0.006	1.5±0.3 (p=0.658)	0.5±0.3 (p=0.05)		
	gamma	18h	80 ppm	Control	0.33±0.03	0.18±0.02	0	0.019±0.002	1.4±0.2	2.1±0.5	1.11±0.06 (p=0.141)	1.00±0.13 (p=0.369)
				BSH	0.37±0.04	0.13±0.06	0	0.028±0.001	1.4±0.2 (p=0.275)	2.3±0.7 (p=0.275)		

Supplementary Table 1. Fitting parameters for the linear-quadratic equation $Y(X) = \exp(-\alpha X - \beta X^2)$, irradiation dose for half-maximal inhibition of viability (IC_{50}), and dose-modifying factor for 20% survival (DMF_{20}) of A172 glioma cells. The parameter β was constrained to non-negative values. Experimental data are expressed as the mean ± the standard deviation (SD). To assess differences between groups (with/without BSH), the Mann – Whitney test was used. One-sample t-test was used to evaluate difference DMF_{20} values from 1.

Cell line	Radiation	Time of incubation	Concentration of BSH	Sample	α		β		IC_{50}, Gy		DMF_{20}	
					AlamarBlue assay	Clonogenic assay	AlamarBlue assay	Clonogenic assay	AlamarBlue assay	Clonogenic assay	AlamarBlue assay	Clonogenic assay
Gl-Tr	proton beam (89.7 MeV)	18h	80 ppm	Control	0.19±0.08	0.17±0.02	0.162±0.002	0.025±0.004	2.3±0.4	2.2±1	1.09±0.16 (p=0.892)	1.18±0.12 (p=0126)
				BSH	0.29±0.07	0.26±0.02	0.005±0.001	0.022±0.005	1.9±0.3 (p=0.08)	1.6±0.7 (p=0.05)		
			160 ppm	Control	-0.03±0.01	0.48±0.03	0.08±0.02	0	2.6±0.7	1.0±0.3	0.98±0.08 (p=0.147)	1.12±0.11 (p=0.178)
				BSH	0.29±0.06	0.51±0.03	0.061±0.007	0	2.0±0.3 (p=0.5)	0.8±0.3 (p=0.127)		
		7h	80 ppm	Control	0.16±0.05	0.21±0.14	0.022±0.002	0.025±0.004	2.4±0.4	1.9±0.8	1.02±0.03 (p=0.566)	1.32±0.15 (p=0.07)
				BSH	0.24±0.05	0.39±0.17	0.009±0.001	0	2.1±0.3 (p=0.268)	1.4±0.6 (p=0.275)		
	proton beam (200 MeV)	18h	80 ppm	Control	-0.03±0.01	0.07±0.02	0.08±0.03	0.058±0.006	2.6±0.7	2.0±1.5	0.92±0.08 (p=0.02)	1.02±0.12 (p=0.415)
				BSH	-0.029±0.002	0.16±0.08	0.06±0.01	0.041±0.009	3.0±0.8 (p=0.05)	1.8±1.5 (p=0.827)		
	gamma	18h	80 ppm	Control	0.005±0.001	0.08±0.01	0.029±0.003	0.023±0.003	4.2±0.2	3.3±1	1.06±0.01 (p=0.008)	1.05±0.13 (p=0.305)
				BSH	0.06±0.02	0.07±0.01	0.026±0.002	0.027±0.003	3.4±0.5 (p=0.05)	3.1±1 (p=0.658)		

Supplementary Table 2. Fitting parameters for the linear-quadratic equation $Y(X) = \exp(-\alpha X - \beta X^2)$, irradiation dose for half-maximal inhibition of viability (IC_{50}), and dose-modifying factor for 20% survival (DMF_{20}) of Gl-Tr glioma cells. The parameter β was constrained to non-negative values. Experimental data are expressed as the mean ± the standard deviation (SD). To assess differences between groups (with/without BSH), the Mann – Whitney test was used. One-sample t-test was used to evaluate difference DMF_{20} values from 1.

Cell line	Radiation	Time of incubation	Cconcentration of BSH	Sample	α		β		IC_{50}, Gy		DMF_{20}	
					AlamarBlue assay	Clonogenic assay	AlamarBlue assay	Clonogenic assay	AlamarBlue assay	Clonogenic assay	AlamarBlue assay	Clonogenic assay
Du145	proton beam (89.7 MeV)	18h	80 ppm	Control	0.11±0.03	0.45±0.01	0.008±0.001	0	5.2±0.5	1.0±0.3	0.95±0.06 (p=0.492)	1.25±0.02 (p=0.05)
				BSH	0.12±0.03	0.57±0.01	0.004±0.001	0	5.1±0.6 (p=0.513)	0.9±0.3 (p=0.275)		
			160 ppm	Control	0.21±0.08	0.21±0.03	0.002±0.001	0.10±0.03	2.3±0.4	1.4±0.3	1.04±0.16 (p=0.351)	0.99±0.08 (p=0.868)
				BSH	0.23±0.07	0.10±0.03	0	0.13±0.05	2.3±0.4 (p=0.827)	1.4±0.4 (p=0.513)		
		7h	80 ppm	Control	0.22±0.05	0.32±0.04	0.013±0.001	0.07±0.02	2.2±0.3	0.84±0.34	1.03±0.01 (p=0.535)	0.99±0.12 (p=0.741)
				BSH	0.21±0.06	0.45±0.06	0.017±0.002	0.028±0.001	2.2±0.3 (p=0.827)	0.78±0.35 (p=0.275)		
			250 ppm, position I	Control	0.21±0.05	0.63±0.02	0.019±0.002	0	2.1±0.7	0.7±0.4	1.08±0.07 (p=0.618)	0.75±0.08 (p=0.005)
				BSH	0.22±0.06	0.48±0.02	0.022±0.002	0	1.9±0.4 (p=0.827)	1.1±0.5 (p=0.275)		
			250 ppm, position II	Control	0.17±0.05	0.34±0.17	0.021±0.017	0.059±0.027	2.4±0.5	1.0±0.4	1.00±0.17 (p=0.541)	1.17±0.12 (p=0.868)
				BSH	0.22±0.05	0.62±0.03	0.013±0.008	0	2.2±0.4 (p=0.513)	0.7±0.3 (p=0.513)		
	Proton beam (200 MeV)	18h	80 ppm	Control	0.013±0.004	0.45±0.02	0.04±0.02	0	4.0±1.2	1.0±0.3	0.98±0.31 (p=0.468)	1.19±0.04 (p=0.144)
				BSH	0.022±0.009	0.58±0.02	0.03±0.01	0	3.9±1.5 (p=0.827)	0.9±0.4 (p=0.127)		
	gamma	18h	80 ppm	Control	0.10±0.05	0.036±0.007	0.013±0.009	0.026±0.003	3.9±0.5	3.9±1.2	0.92±0.2 (p=0.363)	1.00±0.02 (p=0.353)
				BSH	0.17±0.05	0.014±0.005	0.002±0.001	0.029±0.004	3.5±0.6 (p=0.513)	4.0±1.5 (p=0.513)		

Supplementary Table 3. Fitting parameters for the linear-quadratic equation $Y(X) = \exp(-\alpha X-\beta X^2)$, irradiation dose for half-maximal inhibition of viability (IC_{50}), and dose-modifying factor for 20% survival (DMF_{20}) of Du145 cells. The parameter β was constrained to non-negative values. Experimental data are expressed as the mean ± the standard deviation (SD). To assess differences between groups (with/without BSH), the Mann – Whitney test was used. One-sample t-test was used to evaluate difference DMF_{20} values from 1.
